# Editorial: Artificial intelligence in predicting, determining and controlling cell phenotype or tissue function in inflammatory diseases

**DOI:** 10.3389/fimmu.2024.1443534

**Published:** 2024-06-18

**Authors:** Melanie L. Hart, Ryuji Kato, Bernd Rolauffs

**Affiliations:** ^1^ G.E.R.N. Research Center for Tissue Replacement, Regeneration & Neogenesis, Department of Orthopedics and Trauma Surgery, Faculty of Medicine, Medical Center—Albert-Ludwigs-University of Freiburg, Freiburg im Breisgau, Germany; ^2^ Division of Bioscience, Department of Basic Medicinal Sciences, Graduate School of Pharmaceutical Sciences, Nagoya University, Nagoya, Japan; ^3^ Division of Micro-Nano Mechatronics, Institute of Nano-Life-Systems, Institute for Innovation for Future Society, Nagoya University, Nagoya, Japan

**Keywords:** machine learning, deep learning, image processing, predicting, cell phenotype, cell function, tissue function, inflammation

Inflammatory diseases present significant global health challenges, requiring innovative approaches to understand their complexities and develop effective therapies. Our Research Topic, titled “Artificial Intelligence in Predicting, Determining, and Controlling Cell Phenotype or Tissue Function in Inflammatory Diseases”, aimed to leverage the power of artificial intelligence (AI) to address these challenges. We are pleased to announce that seventeen manuscripts were selected from a total of fifty-nine submissions after rigorous reviews. This collection of articles explores various facets of AI-driven analyses, providing valuable insights into immune-related cell signatures in extreme environments, inflammation, degeneration, and inflammatory diseases, as well as the pathogenesis and prediction of disease onset, progression, diagnosis, and therapy. The articles encompass a broad spectrum of inflammatory conditions, including spaceflight, COVID-19, sepsis, cancer, fibrosis, tendinopathy, renal fibrosis, cardiovascular diseases, and stroke. In these articles, a wide range of machine learning/AI methods was utilized, underlining the usefulness and interdisciplinary acceptance of such computational techniques that go well beyond basic statistical methods, e.g., with the focus on identifying/predicting immune-modulation, molecular targets, and single cell phenotype.

## Immune-related cell signatures in extreme environments, inflammation, degeneration and inflammatory diseases

Paralleling the Research Topic’s exploration of immune-related cell signatures, Stratis et al. captured the longitudinal changes in leukocyte transcript levels of astronauts transitioning to and from space, adaptation of leukocyte activity in space, and post-space flight effects using generalized linear modeling, presenting a bridge between statistical methods and machine learning approaches. Their work revealed decreased immune functions when reaching space and increased expression of immune-related genes upon egress back to Earth, shedding light on immuno-modulation in space and longitudinal effects of space on the immune system, highlighting adaptive changes in leukocyte activity in extreme environments.

By harnessing the power of cutting-edge imaging modalities and computational algorithms, Selig et al. and Selig et al. gained unprecedented insights into the dynamic interplay between cellular morphology and inflammation using AI for advanced image analysis and prediction of cell properties. This work proved that image-based features of cell morphology can accurately discriminate between healthy, inflamed, degenerating, and diseased chondrocytes and predict M0, M1-like, M2, M2-like, M2a and M2c macrophage phenotypes with different functions ranging from homeostatic, anti-/pro-inflammatory to anti-fibrotic/fibrotic and tissue repair phenotypes as well as their immunogenic (i.e., cytokine production) potential at the single-cell level. These findings enhance our understanding of cell morphology’s role in inflammation and immune responses, with potential implications for therapeutic strategies in diseases and targeted immunotherapies.

## Disease pathogenesis

Aligning with the Research Topic’s aim of understanding disease pathology and identifying molecular targets in inflammatory diseases, several groups highlight the importance of AI in elucidating cellular and molecular mechanisms underlying inflammatory diseases. By using multi-modal analysis AI, the study by Guo et al. offers new insights of the TNF signaling pathway (FOXO1-PRDX2-TNF axis) in the pathogenesis of tendinopathy during tendon injury and deterioration, which could help in targeting this molecular pathway in treatment of tendinopathy.

Keloids are pathological scars resulting from abnormal wound healing, marked by persistent local inflammation and excessive collagen deposition, with inflammation intensity correlating with scar size. Song et al. identified genes associated with keloid formation using single-cell sequencing and machine learning, revealing that increased glycosphingolipid metabolism activity is linked to fibroblast differentiation and communication, yielding new understandings of this pathway in diagnosis and treatment of keloids. Zhang et al. identified circadian rhythm-related signature genes and their relationship with infiltration of specific immune cell types in development of obstructive sleep apnea (OSA), offering new insights into disease pathogenesis of sleep apnea by the immune system. By identifying common genes, exploring functional pathways, and immune cell infiltration, the studies of Li et al. and Ji et al. shed new light on the interplay between COVID-19 and pericarditis and epilepsy and stress cardiomyopathy, respectively, aligning with the Research Topic's goal of understanding disease pathology through AI-driven analyses and providing new knowledge on the interplay of these conditions. These studies offer new insights into the immune-mediated pathogenesis of disease.

## Prediction of disease onset, progression, diagnosis, and therapy

Aligning with the Research Topic’s aim to advance AI methods for disease diagnosis and prediction in the context of inflammation, Wiffen et al. addressed the pressing need for clinical triage in COVID-19 using multiple biomarker bioprofiling in serum. Through machine learning techniques, this study demonstrated the potential of serum biomarkers in predicting disease severity and triaging patients. Ren et al. developed a diagnostic model for ischemic stroke using nine inflammation-related genes identified through machine learning, which was reflected in immune-related cells in blood samples. Based on machine learning analysis of transcriptome characteristics, Diao et al. identified platelet-related genes that predict poor prognosis in sepsis patients, whereas Zhou et al. dissected the heterogeneity of sepsis by classifying two subclasses of sepsis, adaptive and inflammatory, having distinct immune features and better vs. worse clinical outcomes, which could help recognize patients at high risk of developing sepsis.

Several articles not only identified prognostic disease diagnosis and prediction biomarkers by AI but also used it to understand drug sensitivity and therapeutic effectiveness. Guo et al. developed a diagnostic model for renal fibrosis based on machine learning algorithms by identifying gene biomarkers and the association between these genes and infiltrating immune cells as well as drug sensitivity effects of both established and novel drugs in renal fibrosis. Similarly, Zhang et al. showed the prognostic predictive ability of Snail family transcriptional repressor 2 in various types of cancers, how it relates to infiltration of specific immune cells and effectiveness of a clinical immunotherapy. Coto-Segura et al. used systems biology and quantitative systems pharmacology models to simulate clinical trial-like virtual populations of patients with moderate-to-severe psoriasis treated with various doses of certolizumab, accurately reproducing known biological and clinical activities. Their work identified distinct clusters of virtual patients based on psoriasis-related protein activity and mechanisms of action, explaining differences in drug efficacy among diverse subpopulations clusters and offering new insights into treatment response patient variability influencing therapeutic outcomes. These articles covered various aspects of AI-driven approaches to identify prognostic biomarkers to predict disease progression but also showed how these biomarkers can be used to manage inflammatory diseases and support the use of computational methods as a modelling strategy to explore drug responses, which may reduce and refine pre-clinical and clinical experimentation.

## Summary

Overall, these articles offer a multifaceted perspective on the application of AI in unraveling the intricacies of inflammation and inflammatory diseases. From elucidating molecular mechanisms to predicting disease outcomes and understanding immune modulation in unconventional settings, each contribution brings us closer to a comprehensive understanding of inflammatory pathologies, which would not be possible without harnessing the power of machine learning, computational modeling, and systems biology. We hope that the insights and findings presented in this Research Topic will inspire further research endeavors and foster future efforts aimed at using AI to improve patient outcomes and advance our collective understanding of inflammation in human health and disease.

## Author contributions

MH: Writing – original draft, Writing – review & editing. RK: Writing – review & editing. BR: Writing – review & editing.

